# Chinese holly fruit inhibits melanoma cell proliferation and immune escape by activating P53-P21 signaling and inhibiting JAK2-STAT1 signaling

**DOI:** 10.3389/fnut.2026.1911568

**Published:** 2026-08-17

**Authors:** Liting Meng, Renzhong Zhang, Yuru Wang, Xinyao Zhang, Xinyue Zhang, Mingxiao Zhao, Qiang Chen

**Affiliations:** 1 Department of Biochemistry and Molecular Biology, Health Science Center, Ningbo University, Ningbo, Zhejiang, China; 2 Institute of Drug Discovery Technology, Ningbo University, Ningbo, Zhejiang, China; 3 Ningbo Key Laboratory of Malignant Tumor Immunodiagnosis and Immunotherapy, Ningbo University, Ningbo, Zhejiang, China

**Keywords:** Chinese holly fruit, ilex cornuta lindl. ex paxt fruit, melanoma, p53 signaling, PD-L1

## Abstract

**Introduction:**

Chinese holly (*Ilex cornutaLindl. ex Paxt*) fruit exhibits various biological activities, including heat-clearing and detoxifying, promoting blood circulation to remove stasis, relieving pain and detoxifying, and anti-inflammatory effects. However, its role in inhibiting tumor initiation and progression remains unclear, particularly in melanoma therapy.

**Methods:**

In the present study, a panel of molecular biological approaches, including CCK8 assay, colony formation assay, transwell assay, cell cycle profiling, apoptosis detection, flow cytometry, IHC, Western blot, qPCR, RNAseq and metabonomics were utilized to systematically explore the regulatory effects of CHF on proliferative behaviors and immune evasion potential of melanoma cells.

**Results and Discussion:**

We found that Chinese holly fruit (abbreviated as CHF) extract suppresses the proliferative activity of melanoma by activating the P53-P21 signaling pathway, which downregulates the expression of CDK2 and cyclin family members. Meanwhile, CHF promotes apoptosis by upregulating FAS and GADD45A. Metabolomic profiling revealed that differential metabolites induced by CHF extract treatment were enriched in glucose metabolism, energy metabolism, and the AMPK signaling pathway. Furthermore, CHF extract inhibits PD-L1 expression by suppressing the JAK2-STAT1 signaling pathway, thereby increasing the infiltration of CD3^+^ and CD8^+^T cells into tumor tissues and consequently suppressing melanoma immune escape. More importantly, pharmacological administration of CHF extract improves the anti-tumor efficacy of anti-PD-1/PD-L1 immunotherapy, as demonstrated by enhanced T cell infiltration and slower tumor growth in the combination treatment group compared with monotherapy with either agent. Taken together, these findings highlight a crucial role of CHF extract in anti-melanoma effects, and combination with PD-L1 antibody immunotherapy may be an attractive strategy for melanoma treatment.

## Introduction

Melanoma, the most aggressive type of skin cancer, has seen a rapidly increasing incidence worldwide, posing a serious public health concern (1). Conventional chemotherapy has limited efficacy in the treatment of advanced melanoma, with significant drawbacks. Although breakthrough progress has been made in melanoma therapy over the past decade, the limitations of current approaches remain prominent. For example, combined immunotherapy with CTLA-4 and PD-1 inhibitors can induce durable remission in some melanoma patients, but a considerable proportion of patients still fail to respond to this regimen, and the combination therapy is accompanied by a higher incidence and greater severity of immune-related adverse events (2). Targeted combination therapy for patients with BRAF V600 mutations also faces substantial challenges. Although the strategy of interspersing immune checkpoint inhibition during BRAF/MEK inhibitor therapy exerts a protective effect against brain metastases, low response rates and increased toxicity remain hurdles to be overcome (3, 4). In this context, the present study aims to further optimize therapeutic strategies for melanoma and provide new insights and directions for clinical practice.

Cell cycle arrest in tumor cells serves as a fundamental mechanism to counteract pathological cell proliferation. Notably, the P53-P21 signaling cascade acts as a central regulatory hub, governing multiple cellular processes including cell cycle arrest, apoptosis, senescence, and the preservation of genome integrity (5, 6). Studies have shown that cytoplasmic P53 can directly translocate to mitochondria and interact with Bcl-2 family proteins, thereby activating the intrinsic apoptotic pathway independently of P21-dependent transcriptional pathways (7). Meanwhile, activated P53 functions as a transcription factor that binds to the promoter region of the *CDKN1A* gene and efficiently induces P21 protein expression. P21, as a cyclin-dependent kinase inhibitor, binds to and inhibits the activity of Cyclin E/CDK2 and Cyclin D/CDK4/6 complexes (8, 9). Furthermore, CD8^+^ T cell-mediated specific immune responses represent an important mechanism for eliminating abnormal cells. The JAK-STAT signaling can be activated by cytokines such as IFNγ, inducing the expression of the immunosuppressive molecule PD-L1 on tumor cells to evade immune system surveillance (10, 11). Anti-PD-L1/PD-1 therapy has shown significant and durable efficacy in the treatment of melanoma, in which nivolumab and pembrolizumab have been demonstrated in multiple studies to improve the prognosis of melanoma patients (12, 13) and were approved by the FDA in 2015 as first-line therapy for advanced melanoma. However, these inhibitors still face challenges including inconsistent efficacy, obvious side effects, and susceptibility to resistance (14). Therefore, improving the efficacy of immune checkpoint blockade, reducing toxicity, and overcoming resistance are key challenges that need to be addressed in melanoma immunotherapy.

Chinese holly fruit (CHF), a traditional Chinese medicine, is the mature fruit of the Ilex cornuta plant from the Aquifoliaceae family. Its major chemical components include flavonoids, triterpenoids, saponins, polysaccharides, and alkaloids (15). It is known for nourishing the liver and kidney, strengthening bones and muscles, and astringing the lower energizer, and is used for conditions such as liver and kidney deficiency, spermatorrhea, leukorrhea, and diarrhea. Modern pharmacological studies have shown that CHF also possesses anti-inflammatory, antioxidant, hypoglycemic, and hepatoprotective effects (16). The active ingredients in CHF exhibit anti-tumor potential. For example, alkaloids exert anticancer effects by regulating the PI3K/Akt/mTOR (PAMT) pathway (17); flavonoids inhibit EGFR/MAPK and PI3K/Akt/mTOR signaling pathways that are key to cancer cell growth, thereby suppressing tumor cell division and growth (18). In this study, we found that CHF inhibits the proliferation, migration, and invasion of melanoma cells, upregulates the expression of the tumor suppressor P21 to downregulate downstream cyclins such as Cyclin B1 and Cyclin E2, and consequently suppresses the proliferative activity of melanoma. Furthermore, CHF downregulates PD-L1 expression by inhibiting the JAK2/STAT1 signaling pathway and pharmacologically enhances the anti-tumor efficacy of anti-PD-1/PD-L1 immunotherapy, suggesting its potential as an immune activator.

## Methods

### Mice and cell

C57BL/6 mice were obtained from the Laboratory Animal Center of Ningbo University. All mice were maintained under specific pathogen-free conditions with a 12:12-h light/dark cycle and received a regular chow diet and water ad libitum. Six- to eight-week-old mice were used in all animal experiments and were randomly divided into groups. All experimental procedures involving animals were conducted following animal protocols approved by the Laboratory Animal Center of Ningbo University.

The murine melanoma cell lines (B16) and human melanoma cell lines (A375) were purchased from Cell Bank of Type Culture Collection of Chinese Academy of Sciences (Shanghai, China). The cell lines were characterized by HySigen Biotechnology Co., Ltd (Suzhou, China) using short tandem repeat markers. B16 and A375 were maintained in DMEM medium (L120KJ, Basalmedia Technologies) supplemented with 10% fetal bovine serum (FBS; C04001, VivaCell) and 100 U/mL penicillin-streptomycin. All cells were tested negative for mycoplasma contamination using the single-step PCR method and were maintained in a humidified chamber with 5% CO_2_ at 37 °C.

### Cell viability, migration, and invasion assays

The analysis was performed as described previously (19). Cell viability analysis used the CCK8 kit (GK10001, GLPBIO); invasion assays were performed using Corning inserts coated with matrigel (354230, Corning). Migration assays were performed with similar procedures but using Corning inserts without matrigel. For each experiment, the number of cells in random fields (400 × ) was documented.

### Tumor growth in mice

A total of 2 × 10^6^ B16 cells were subcutaneously injected into the dorsal flanks of randomly selected C57BL/6 mice or nude mice. Tumor size was measured every 2 days from the 5th day after inoculation. The volume of the tumor was calculated using the following formula: Volume = Length × Width^2^ × 0.52. At the end of the experiment, mice were euthanized via gradual-fill 100% medical-grade CO_2_ in accordance with the AVMA Guidelines for the Euthanasia of Animals (2020 Edition). And tumor tissues were harvested for weight measurement and further analyses. The Extract of Chinese holly fruit was given intragastrically every day (50 mg/kg), and PD-1 (BE0273, BioXcell) or PD-L1 antibody (BE0101, BioXcell) was intraperitoneally injected every 2 days (5 mg/kg) since the 5th day of tumor cell inoculation. Isotype IgG were used as controls.

### Extract of Chinese holly fruit

Select Chinese holly fruits free of mold spots and black blemishes, and transfer them into 2 mL grinding tubes. Add 1 mL of solvent (ultrapure water) per tube. Homogenize thoroughly using a high-throughput tissue grinder (JCZYM-24R, Zhejiang Jiechen Instrument Equipment Co., Ltd.) at 4 °C, 60 Hz for 30 s, followed by a 30-s pause; repeat the cycle 30–60 times depending on tissue disruption. After complete homogenization, centrifuge at 4 °C, 13,000g for 10 min, collect the supernatants, pool and mix well. Repeat centrifugation and supernatant collection. Remove the solvent using a lyophilizer concentrator. Determine the weight difference before and after concentration using an analytical balance, and adjust the final solution concentration to 10 mg/mL. The dose range of CHF applied in both *in vitro* cellular assays and *in vivo* animal experiments in the present study was rigorously determined through a combination of empirical evidence from previously published relevant studies (20) and the pre-experimental data. The stock solution was applied to cellular experiments at a 1:100 dilution ratio, with a final working concentration of 100 μg/mL.

### Metabolomics analysis

#### Sample preparation

The cell sample stored at −80 °C refrigerator was thawed on ice. Then vortexes for 10 s and mixed well. Take the mixed sample and place it in a 2 mL EP tube. Add 500 μL of 70% methanol with internal standard extract and vortex for 3 min. Place the centrifuge tube with samples in liquid nitrogen for 5 min, remove and thaw on ice for 5 min, vortex for 2 min and mix well. Centrifuge at 4 °C, 12,000 r/min for 10 min, remove 300 μL of supernatant into another corresponding numbered centrifuge tube, and let stand in the refrigerator at −20 °C for 30 min. Centrifuge at 4 °C for 3 min at 12,000 r/min, and transfer the supernatant to the liner tube of the corresponding injection vial for UPLC-MS/MS analysis.

#### UPLC-MS/MS analysis

The sample extracts were analyzed using an UPLC-ESI-MS/MS system (UPLC, ExionLC™ AD, https://sciex.com.cn/) and Tandem mass spectrometry system (https://sciex.com.cn/). The analytical conditions were as follows, UPLC: column, Agilent SB-C18 (1.8 μm, 2.1 mm × 100 mm); The mobile phase was consisted of solvent A, pure water with 0.1% formic acid, and solvent B, acetonitrile with 0.1% formic acid. Sample measurements were performed with a gradient program that employed the starting conditions of 95% A, 5% B. Within 9 min, a linear gradient to 5% A, 95% B was programmed, and a composition of 5% A, 95% B was kept for 1 min. Subsequently, a composition of 95% A, 5.0% B was adjusted within 1.1 min and kept for 2.9 min. The flow velocity was set as 0.35 mL per min; the column oven was set to 40 °C; The injection volume was 2 μL. The effluent was alternatively connected to an ESI-triple quadrupole-linear ion trap (QTRAP)-MS. The ESI source operation parameters were as follows: source temperature 500 °C; ion spray voltage (IS) 5500 V (positive ion mode)/−4500 V (negative ion mode); ion source gas I (GSI), gas II(GSII), curtain gas (CUR) were set at 50, 60, and 25 psi, respectively.

#### Cluster, differential metabolites and KEGG analysis

The HCA (hierarchical cluster analysis) results of samples and metabolites were presented as heatmaps with dendrograms, while pearson correlation coefficients (PCC) between samples were calculated by the corrplot (v0.92) package in R. Both HCA and PCC were carried out by R package ComplexHeatmap (v 2.9.4). Differential metabolites were determined by VIP (Variable Importance in Projection, VIP > 1) and *P*-value (*P*-value < 0.05, Student's *t* test). VIP values were extracted from OPLS-DA result, which also contain score plots and permutation plots, was generated using R package MetaboAnalystR (v1.0.1). Identified metabolites were annotated using KEGG Compound database (http://www.kegg.jp/kegg/compound/), annotated metabolites were then mapped to KEGG Pathway database (http://www.kegg.jp/kegg/pathway.html).

### RNA-seq analysis

Total RNA was extracted from the control and CHF treatment groups using Trizol (Invitrogen, Carlsbad, CA, USA) according to manual instruction, and 50μL of DEPC-treated water was added to dissolve the RNA. Subsequently, total RNA was qualified and quantified using a Nano Drop and Agilent 2100 bioanalyzer (Thermo Fisher Scientific, MA, USA). Oligo(dT)-attached magnetic beads were used to purified mRNA, and fragmented into small pieces using fragmentation reagent. First-strand cDNA was generated using random hexamer-primed reverse transcription, followed by a second-strand cDNA synthesis. The synthesized double-stranded cDNA was subjected to end-repair and 3′ adenylated, and adapters were added to the ends of these cDNA fragments. Amplified the cDNA fragments, and the PCR products were purified with AMPure XP Beads. Fragment size and concentration were determined using the Agilent 2100 Bioanalyzer. Denatured the PCR product to single-stranded, and circularized by the splint oligo sequence to obtain the final library. The library was amplified with phi29 to make DNA nanoball (DNB) and load into the patterned nanoarray, thereby sequencing by combinatorial Probe-Anchor Synthesis (cPAS) (BGI-Shenzhen, China). The heatmap was drawn by pheatmap (v1.0.8) according to the gene expression in different samples. Essentially, differential expression analysis was performed using the DESeq2 (v1.4.5) with *Q* value ≤ 0.05. To gain insight to the change of phenotype, KEGG (https://www.kegg.jp/) enrichment analysis of annotated different expressed gene was performed by Phyper (https://en.wikipedia.org/wiki/Hypergeometric_distribution) based on Hypergeometric test. The significant levels of terms and pathways were corrected by *Q* value with a rigorous threshold (*Q* value ≤ 0.05) by Bonferroni. The data of RNA-seq has been deposited in a public repository.

### Real-time quantitative PCR (RT-qPCR)

Total RNA of cells and tissues were purified with TRIzol and extracted according to the manufacturer's instructions (CW0584S, CWBIO). Total RNA (2μg) was used to synthesize the cDNA using RT Master Mix (G490, ABM). RT-qPCR was performed by Genious 2X SYBR Green Fast qPCR Mix (RK21204, ABclonal) using Roche LC480 II system. The sequences of qPCR primers were listed as follows:

h-β-actin, Forward 5′-CATGTACGTTGCTATCCAGGC-3′, Reverse 5′-CTCCTTAATGTCACGCACGAT-3′; h-Cyclin B1, Forward 5′-ATACTGCCTCTCCAAGCCCAATG-3′, Reverse 5′-TTGCTCTTCCTCAAGTTGTCTCAGA-3′; h-Cyclin B2, Forward 5′-GCTGGTACAAGTCCACTCCAAGTT-3′, Reverse 5′-AAGCCAAGAGCAGAGCAGTAATCC-3′; h-Cyclin E2, Forward 5′-GTCCTGTAACAATCATCTCCTGGCT-3′, Reverse 5′-CACAAGGCAGCAGCAGTCAGT-3′; h-P53, Forward 5′-CCATCTACAAGCAGTCACAGCACAT-3′, Reverse 5′-GCTCATAGGGCACCACCACACTA-3′; h-P21, Forward 5′-CATGTGGACCTGTCACTGTCTTGT-3′, Reverse 5′-CGGCGTTTGGAGTGGTAGAAATCT-3′; h-CDK2, Forward 5′-CCAGAAACAAGTTGACGGGAGAGG-3′, Reverse 5′-CCAGTGAGAGCAGAGGCATCCA-3′; h-FAS, Forward 5′-TGAAGGACATGGCTTAGAAGTGGAA-3′, Reverse 5′-ACTTGGTGTTGCTGGTGAGTGT-3′; h-GADD45A, Forward 5′-TCTCGGCTGGAGAGCAGAAGAC-3′, Reverse 5′-GCCACATCTCTGTCGTCGTCCT-3′; h-JAK2, Forward 5′-TCAGAGAAGAAGACAGGAAGACAGG-3′, Reverse 5′-TGAGAATCCAGAGCACTTAGAGGTT-3′; h-STAT1, Forward 5′-GCAGAACAGAGAACACGAGACCAAT-3′, Reverse 5′-CTGTCTCCGCTTCCACTCCACTA-3′; h-PD-L1, Forward 5′-GCTATGGTGGTGCCGACTACAA-3′, Reverse 5′-TGGAATTGGTGGTGGTGGTCTT-3′; m-β-actin, Forward 5′-ACTATTGGCAACGAGCGGTTCC-3′, Reverse 5′-GGCATAGAGGTCTTTACGGATGTCA-3′; m-Cyclin B1, Forward 5′-CTGAGCCTGAACCTGAACTTGAACA-3′, Reverse 5′-CCATCATCTGCGTCTACGTCACTC-3′; m-Cyclin B2, Forward 5′-CGAGGACATAGATAACGAGGACAGG-3′, Reverse 5′-ACCAGGATGGCACGCATACG-3′; m-Cyclin E2, Forward 5′-GCTGCTGCCGCCTTATGTCATT-3′, Reverse 5′-GCACCATCCAGTCTACACATTCCG-3′; m-P53, Forward 5′-CGGCTCTGAGTATACCACCATCCA-3′, Reverse 5′- TTCTTCTTCTGTACGGCGGTCTCT-3′; m-P21, Forward 5′-ATATCCAGACATTCAGAGCCACAGG-3′, Reverse 5′-CGAAGTCAAAGTTCCACCGTTCTC-3′; m-CDK2, Forward 5′-CCGCTGTGCTCCTATCTA-3′, Reverse 5′-GTTGGTCAATCTCAGAATCTC-3′; m-FAS, Forward 5′-TACTGCGATTCTCCTGGCTGTGA-3′, Reverse 5′-CCATAGGCGATTTCTGGGACTTTGT-3′; m-GADD45A, Forward 5′-GCAGAGCAGAAGACCGAAAGGATG-3′, Reverse 5′-CAGCAGGCACAGTACCACGTTATC-3′; m-JAK2, Forward 5′-GCAGCAGCAGAACCTACAGATACG-3′, Reverse 5′-CTCATCATGTCTAACACCGCCATCC-3′; m-STAT1, Forward 5′-CTGCTGTGCCTCTGGAATGATGG-3′, Reverse 5′-GGAAGTCAGGTTCACCTCCGTTCT-3′; m-PD-L1, Forward 5′-GTGGTGCGGACTACAAGCGAAT-3′, Reverse 5′-ACGGGTTGGTGGTCACTGTTTG-3′; m-IL-1β, Forward 5′-GCACTACAGGCTCCGAGATGAA-3′, Reverse 5′-GTCGTTGCTTGGTTCTCCTTGT-3′; m-IFNγ, Forward 5′-ACTCAAGTGGCATAGATGTGGAAGA-3′, Reverse 5′-ATGACGCTTATGTTGTTGCTGATGG-3′; m-IL-2, Forward 5′-TGGACCTCTGCGGCATGTTCT-3′, Reverse 5′-CCACCACAGTTGCTGACTCATCAT-3′; m-TNFα, Forward 5′-GCCACCACGCTCTTCTGTCTAC-3′, Reverse 5′-GGCTACAGGCTTGTCACTCGAATT-3′;

### Western blot and flow cytometry analysis

Cells or tissues were lysed with RIPA buffer supplemented with protease inhibitor cocktail and phosphatase inhibitors. The protein concentrations of lysates were quantified by BCA protein quantitative method. Equal quality protein (20μg) was separated by SDS-PAGE, and transferred to a PVDF membrane (10600023, Cytiva). The PVDF membrane was cut according to protein molecular weight and incubated with the corresponding primary antibodies overnight at 4 °C. Then membranes were incubated with secondary antibodies. The signal was detected using ECL blotting substrates (RM02867, ABclonal) and captured by a chemiluminescence imager (Tanon, China). The primary antibodies used in Western blot were listed as follows: anti-Cyclin B1 (Cat# A22435, ABclonal), anti-h-PD-L1 (Cat# 13684, CST), anti-m-PD-L1 (Cat# 60475, CST), anti-β-actin (Cat# TA-09, ZSGB-BIO), anti-Cyclin B2 (Cat# 216441-AP, Proteintech), anti-Cyclin E2 (Cat# 11935-1-AP, Proteintech), anti-P53 (Cat# A0263, ABclonal), anti-P21 (Cat# A19094, ABclonal), anti-CDK2 (Cat# A0294, ABclonal), anti-STAT1 (Cat# 14994, CST), anti-JAK2 (Cat# A19629, ABclonal), anti-pSTAT1Y^701^ (Cat# 9167, CST).

Flow cytometry analysis and cell cycle assay was performed as described previously (21). Cells underwent extracellular PD-L1 staining with corresponding fluorophore conjugated antibody per million cells in 100 μL volume. The antibodies were list as follows: anti-h-PD-L1-PE (Cat# 329706, Biolegend), anti-m-PD-L1-PE/Cy7 (Cat# 155406, Biolegend). Isotype IgG or unstained cells were used as controls. Samples were analyzed by Beckman CytExpert and Flowjo X software.

### Immunohistochemistry and TUNEL analysis

Tissue with 4% formalin fixation and their chips with paraffin embedding were cut at 5 μm, followed by immunohistochemistry (IHC) staining according to directions of IHC kit (SP9000, ZSGB-BIO) and were incubated with primary and biotinylated secondary antibodies, respectively. The signal was detected using HRP conjugated streptavidin with the chromogenic substrate DAB. The specific antibodies used in this experiment were listed as follows: anti-m-CD3 (Cat# 99940, CST), anti-m-CD8 (Cat# 98941, CST), anti-Ki67 (Cat# A20018, ABclonal). TUNEL analysis was performed according to the manufacturer's instructions (BRK5877, ABclonal).

### Statistical analysis

Statistical analyses were performed with GraphPad Prism 8.0 software (San Diego, USA). Quantitative data were normally distributed and presented as mean ± SD. Statistically significant differences (*P* < 0.05) were examined using independent-Samples *t*-test, one-way or two-way ANOVA.

## Results

### CHF suppresses the proliferation of melanoma cells *in vitro* and *in vivo*

**Figure 1:**
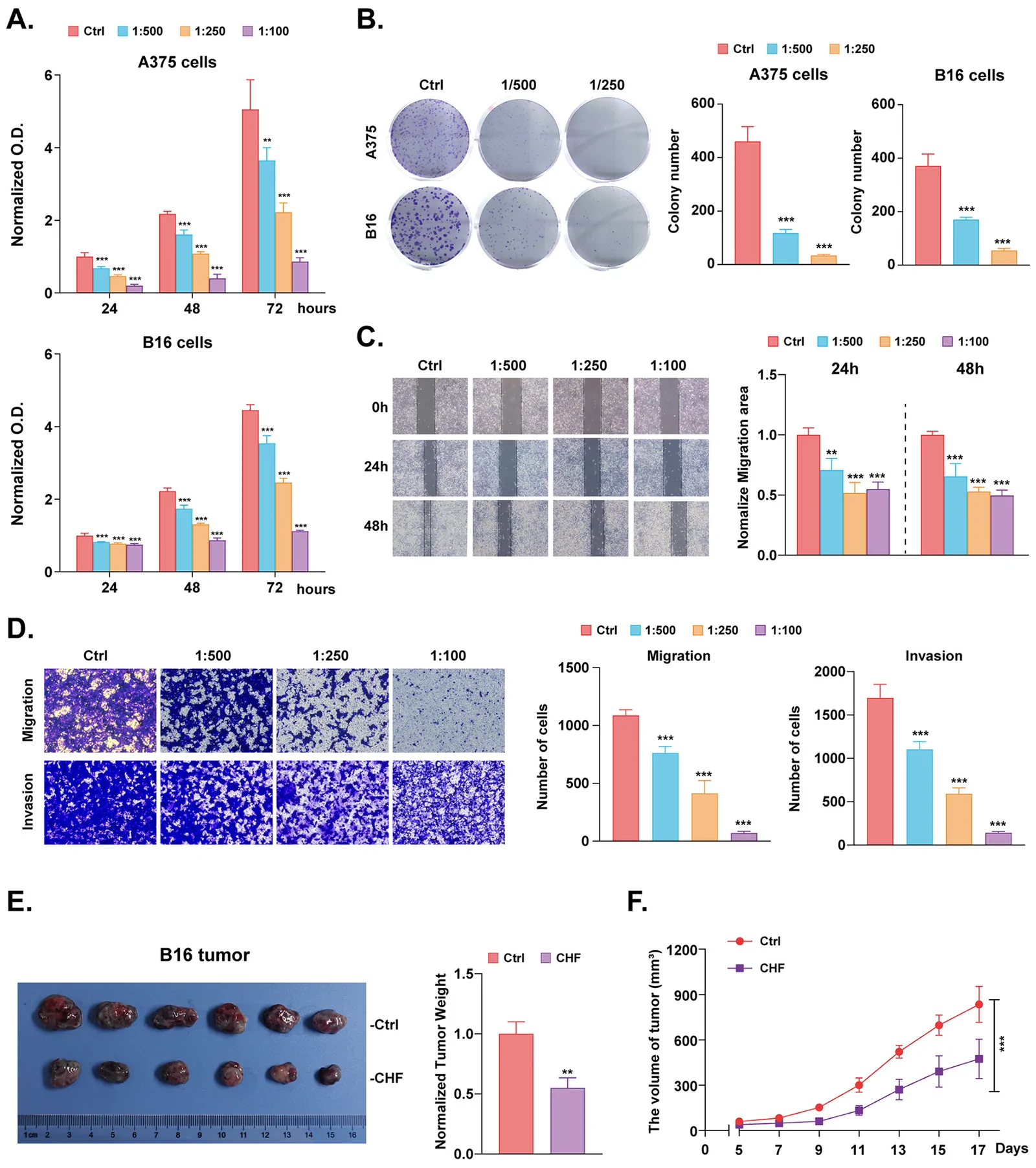
CHF suppresses the proliferation of melanoma cells *in vitro* and *in vivo*. **(A, B)** CHF inhibits the proliferative vitality of melanoma cells *in vitro* (*n* = 3). **(C, D)** CHF suppresses the migration and invasion ability of melanoma cells (*n* = 3). **(E, F)** CHF significantly inhibits the growth of melanoma in mice (*n* = 6). Data are presented as means ± SD. **p < 0.01, ***p < 0.001, based on independent-samples *t*-test or one-way ANOVA.

To investigate the effect of CHF on the proliferation vitality of melanoma cells, we treated mouse and human melanoma cells (B16 and A375) with gradient concentrations of CHF. CCK8 assays revealed that CHF inhibited the proliferation vitality of B16 and A375 cells *in vitro* in a dose-dependent manner (Figure 1A). Colony formation assays showed that treatment with different concentrations of CHF significantly suppressed the colony-forming ability of both A375 and B16 cells compared with the control group (Figure 1B). Furthermore, wound healing and transwell assays were performed to investigate the effect of CHF on the migration and invasion of melanoma cells. Wound healing assay results demonstrated that the wound closure ability of cells in each treatment group was markedly reduced compared with the control group (Figure 1C); and the transwell assay results showed that after 24 h of treatment with different concentrations of CHF, the number of melanoma cells in the lower chamber was significantly decreased in both migration and invasion assays (Figure 1D). These results indicate that CHF has a potential inhibitory effect on the proliferation, migration, and invasion of melanoma cells. Nevertheless, its effect on melanoma growth *in vivo* remains unclear. Therefore, a xenograft tumor model was employed to investigate whether CHF exerts an inhibitory effect on melanoma *in vivo*. We inoculated B16 cells into C57BL/6 mice and monitored tumor growth, and the comparison of final tumor volumes and normalized tumor weights revealed that CHF treatment significantly reduced both xenograft tumor weight and growth in B16 tumor-bearing mice compared to that of the control group (Figures 1E, F). In addition, we also found that compared with the control group, CHF treatment had no significant effect on the body weight of mice (Supplementary Figure 1A), and had no significant effect on important organs such as spleen, kidney, and liver, as evaluated by organ morphology and HE staining (Supplementary Figures 1B–D), indicating that CHF did not have potential toxicity to mice. Collectively, these results demonstrate that CHF persistently suppresses the growth of melanoma cells *in vitro* and *in vivo*, exhibiting reliable anti-tumor activity.

**Figure 2:**
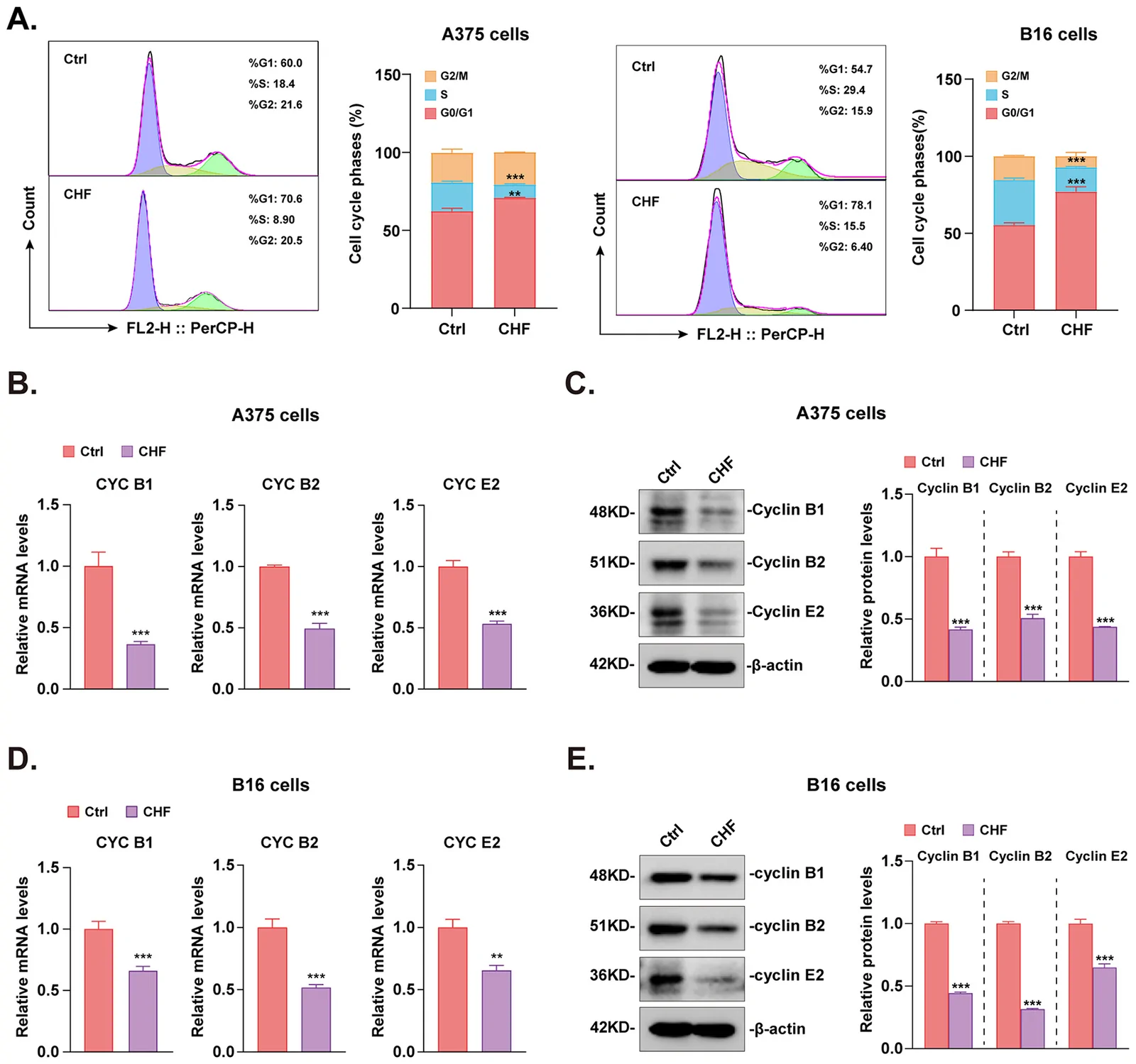
CHF regulates cyclin expression to suppress the proliferative vitality of melanoma cells. **(A)** CHF arrests the cell cycle of melanoma cells (*n* = 3). **(B–E)** CHF significantly inhibits the mRNA **(B, D)** and protein **(C, E)** levels of Cyclin B1, Cyclin B2, and Cyclin E2 (*n* = 3). Data are presented as means ± SD. **p < 0.01, ***p < 0.001, based on independent-samples *t*-test.

### CHF may regulate cyclin expression to suppress melanoma cell activity

Given that cell proliferation is closely associated with the cell cycle, we hypothesized that CHF inhibits melanoma proliferation by affecting cell cycle progression. To test this, flow cytometry was performed to examine the effect of CHF on the cell cycle of A375 and B16 cells. Compared with the control group, the CHF treatment group exhibited a markedly decreased proportion of cells in the S phase and a significantly increased proportion of cells in the G0/G1 phase (Figure 2A), suggesting that CHF may arrest cells at the G0/G1 phase by inhibiting DNA synthesis, thereby preventing their entry into the S phase. Various cyclins are closely related to cell proliferation and cell cycle regulation. For instance, *B*-type cyclins serve as key regulators of mitotic entry (M phase) and chromosome segregation, whereas E-type cyclins govern the G1/S transition (DNA synthesis phase) (22). To explore the potential molecular mechanisms by which CHF affects the melanoma cell cycle, we examined the expression of several cyclins (Cyclin B1, Cyclin B2, and Cyclin E2) in A375 and B16 cells using RT-qPCR and Western blot to assess mRNA and protein levels, respectively. The results showed that CHF significantly downregulated Cyclin B1, Cyclin B2, and Cyclin E2 at both mRNA and protein levels (Figures 2B–E). These results indicate that CHF may affect the cell cycle by inhibiting the expression of Cyclin B1, Cyclin B2, and Cyclin E2, thereby suppressing the proliferative activity of melanoma cells.

### CHF promotes apoptosis in melanoma cells

**Figure 3:**
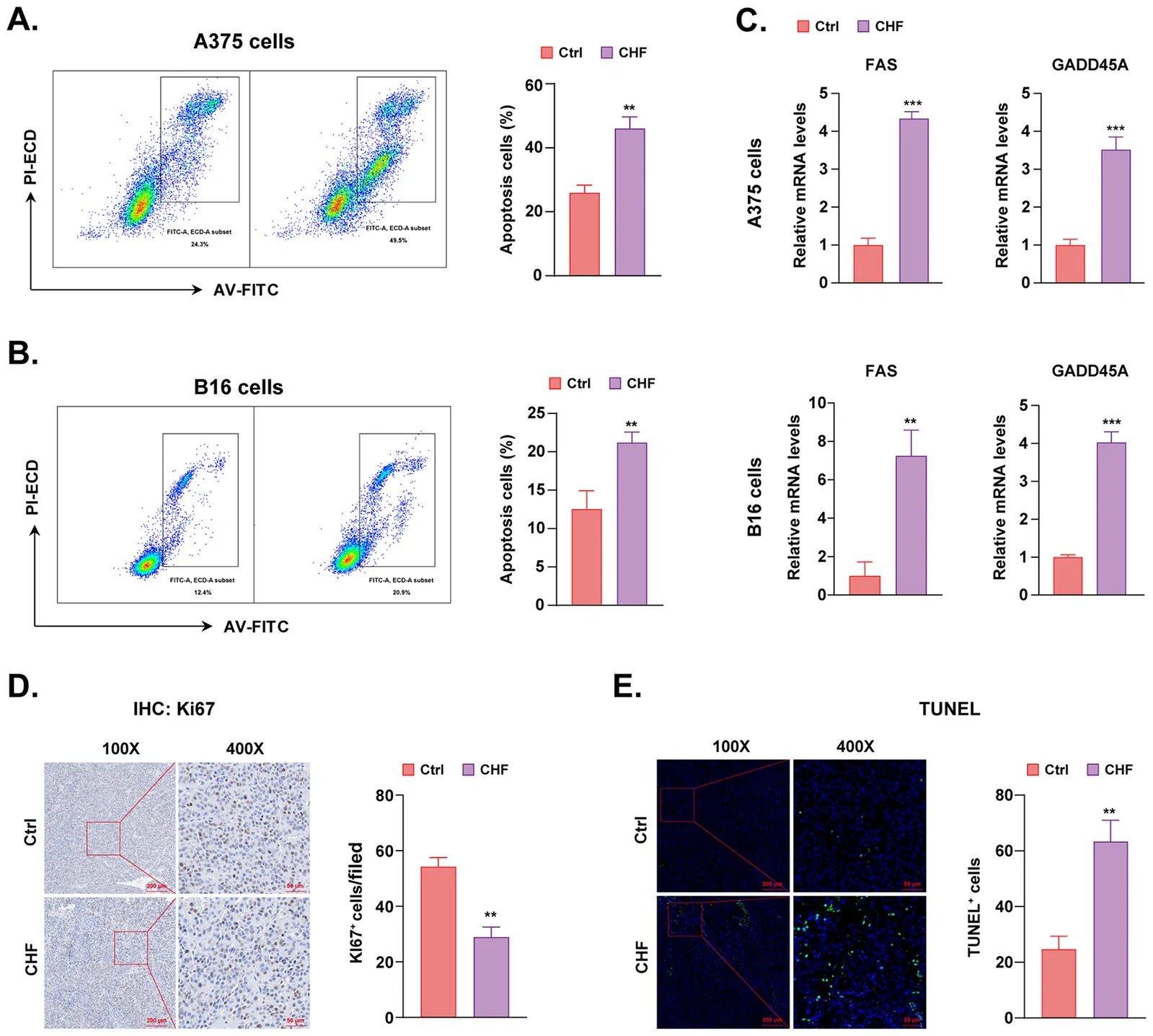
CHF promotes apoptosis in melanoma cells. **(A, B)**. CHF significantly promotes apoptosis of melanoma cells (*n* = 3). **(C)** CHF upregulates the expression of the apoptosis-related genes *FAS* and *GADD45A* (*n* = 3). **(D)** The percentage of Ki67-positive cells in tumor tissues is lower in the CHF-treated tumor tissues (*n* = 3). **(E)** The number of TUNEL-positive cells is significantly increased in CHF-treated tumor tissues (*n* = 3). Data are presented as means ± SD. **p < 0.01, ***p < 0.001, based on independent-samples *t*-test.

Activation of the P53-P21 signaling pathway is frequently coupled with pro-apoptotic signaling. To investigate whether CHF induces apoptosis in melanoma cells, we further examined its effect on apoptosis in A375 and B16 cells by flow cytometry. Compared with the control group, the apoptosis rate was significantly increased in CHF-treated cells (Figures 3A, B). To explore whether CHF activates the extrinsic apoptotic pathway, we examined the expression of FAS, a key death receptor in this pathway. Upon binding to its ligand FASL, FAS recruits the adapter protein FADD and Caspase-8 to form the death-inducing signaling complex (DISC), which subsequently activates the caspase cascade (23) and ultimately induces apoptosis. GADD45A is a “molecular sentinel” with multiple regulatory functions, involved in various signaling pathways and cellular processes. Its expression is critically regulated by the tumor suppressor P53: upon DNA damage, P53 directly binds to the promoter region of the GADD45A gene and strongly induces its transcriptional expression (24). Therefore, we performed RT-qPCR assays to determine whether CHF promotes apoptosis in A375 and B16 cells by affecting the transcriptional levels of FAS and GADD45A. The results showed that, compared with the control group, the mRNA levels of FAS and GADD45A were significantly elevated in the CHF-treated group (Figure 3C). Collectively, these results indicate that CHF may promote apoptosis in melanoma cells by inducing the expression of the apoptotic molecules FAS and GADD45A. Furthermore, we performed Ki67 and TUNEL staining on tumor tissues harvested from the xenograft tumor model. Ki67 staining results showed that the number of Ki67-positive cells in tumor tissues from CHF-treated mice was significantly lower than that in the control group (Figure 3D), indicating that tumor proliferative activity was markedly reduced in the treatment group. TUNEL staining results showed that the number of TUNEL-positive cells in CHF-treated tumor tissues was significantly increased compared with the control group (Figure 3E). These results collectively demonstrate that CHF suppresses melanoma proliferation *in vivo* while promoting tumor cell apoptosis.

### CHF promotes apoptosis and arrests the cell cycle of melanoma by activating the P53-P21-CDK2 pathway

**Figure 4:**
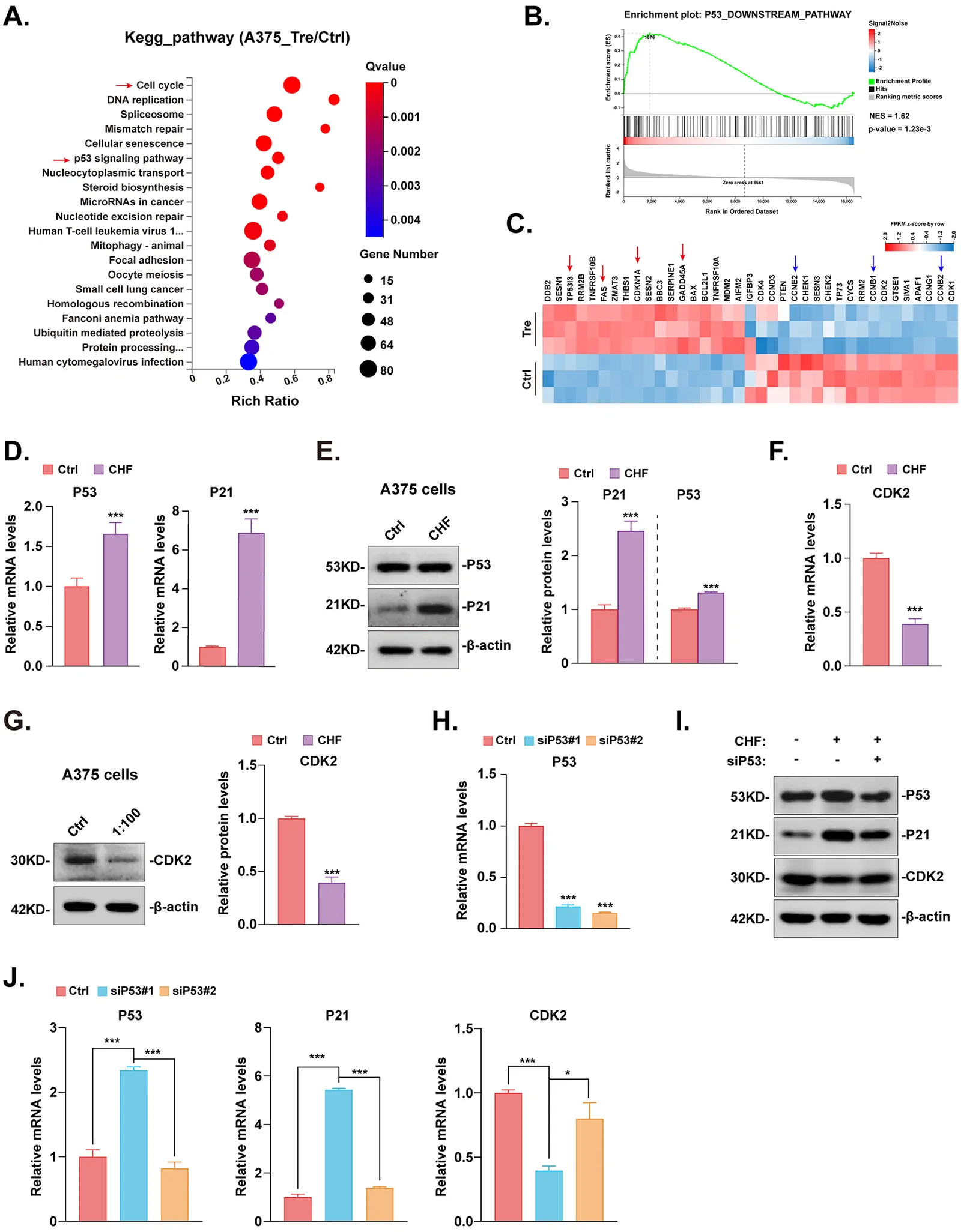
CHF promotes apoptosis and arrests the cell cycle of melanoma by activating the P53-P21-CDK2 pathway. **(A, B)** RNA-seq analysis revealed that the P53 signaling pathway was significantly altered in CHF-treated cells. **(C)** Genes involved in cell cycle regulation and apoptosis were significantly changed. **(D–G)** Both P53 and P21 were markedly upregulated and CDK2 was significantly downregulated in CHF-treated melanoma cells (*n* = 3). **(H)** Evaluation of the efficiency of siRNA targeting P53 (*n* = 3). **(I, J)** Knockdown P53 significantly attenuated the upregulation of P21 induced by CHF and partially rescued the expression of CDK2 (*n* = 3). Data are presented as means ± SD. *p < 0.05, **p < 0.01, ***p < 0.001, based on independent-samples *t*-test or one-way ANOVA.

As described above, our studies have demonstrated that CHF attenuates the proliferative activity of melanoma cells *in vitro* by affecting the expression of key cyclins. However, the mechanism by which CHF affects cyclins remains unclear. Therefore, we performed RNA-seq analysis to preliminarily identify the potential signaling pathways involved. KEGG enrichment analysis revealed that genes related to the P53 signaling pathway were significantly enriched (Figure 4A), and genes involved in cell cycle regulation (e.g., *CCNE2, CCNB1*, and *CCNB2*) or mediating apoptosis genes (e.g., *FAS* and *GADD45A*) were activated (Figures 4B, C). These results suggest that CHF promotes apoptosis and inhibits the cell cycle of melanoma cells by activating the P53-P21-CDK2 signaling pathway. To verify this, we examined the mRNA and protein levels of the relevant target genes in A375 and B16 cells by RT-qPCR and Western blot, respectively. The results showed that, compared with the control group, the mRNA and protein levels of P53 and P21 were significantly upregulated (Figures 4D, E, Supplementary Figures S2A, B), and the expression of CDK2 was inhibited in CHF-treated cells (Figures 4F, G, Supplementary Figures S2C, D). Because the P53-P21-CDK2 pathway serves as a core brake system that determines whether to pause the cell cycle for repair in response to stress signals such as DNA damage, when P21 is induced and abundantly expressed by P53, it inhibits the kinase activity of CDK2, thus leading to cell cycle arrest at the G1 phase and preventing entry into the S phase (25, 26). To determine the role of P53 in CHF mediated anti-melanoma, an experiment utilizing P53-targeting small interfering RNA (siRNA) was employed (Figure 4H). And we found that knockdown P53 significantly attenuated the upregulation of P21 induced by CHF and partially rescued the expression of CDK2 (Figures 4I, J), confirming the central role of the P53-P21 pathway in the antitumor activity of CHF.

### CHF downregulates PD-L1 expression by inhibiting JAK2-STAT1 signaling

**Figure 5:**
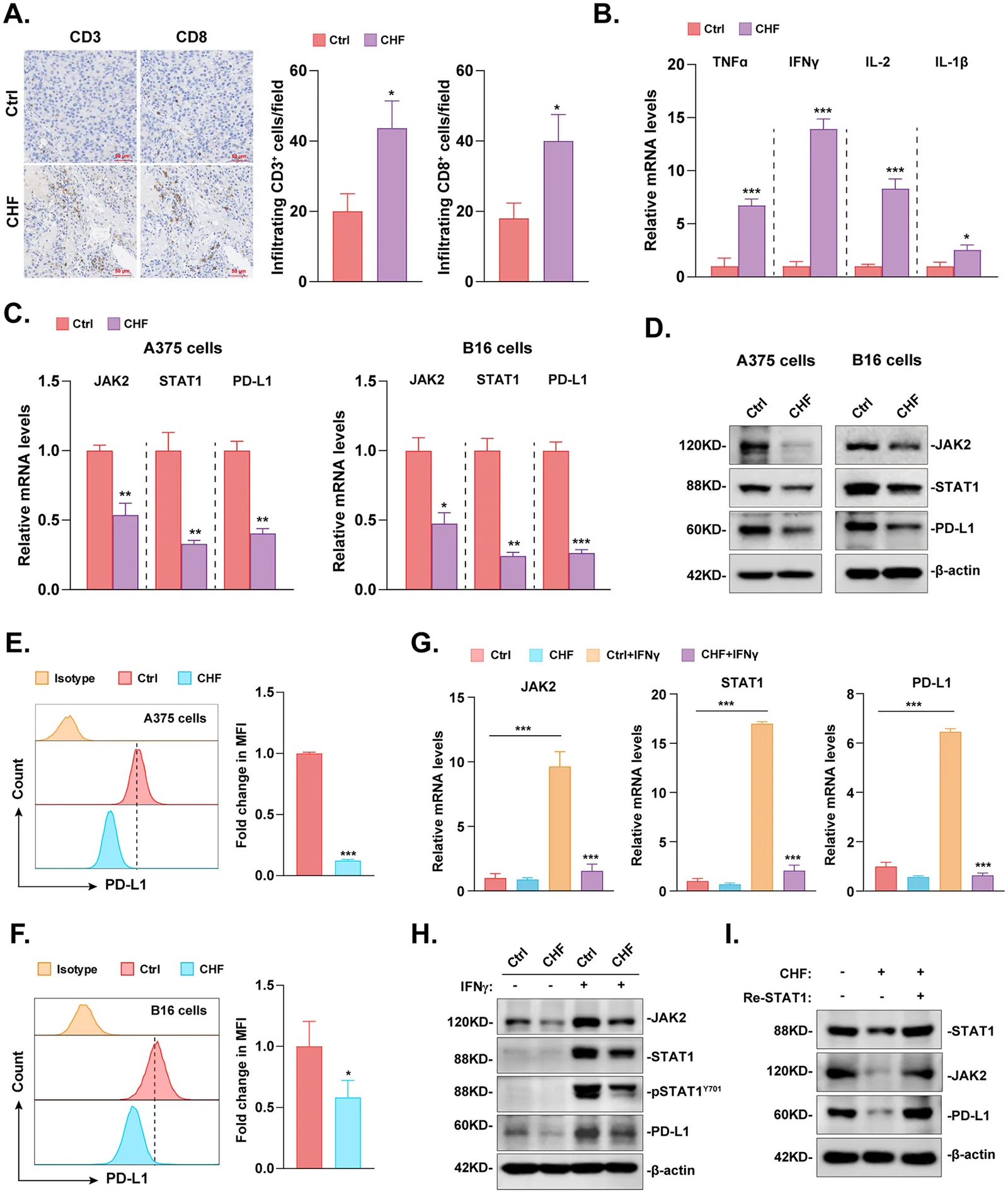
CHF downregulates PD-L1 expression by inhibiting JAK2-STAT1 signaling. **(A)** The number of infiltrating CD3^+^ and CD8^+^ T cells in tumor tissues is significantly increased in the CHF-treated tumor tissues. **(B)** The expression profiles of TNFα, IFNγ, IL-2, and IL-1β in the tumor tissues. **(C, D)** CHF significantly inhibits the mRNA and protein levels of JAK2, STAT1, and PD-L1 (*n* = 3). **(E, F)** CHF significantly reduces PD-L1 levels on the surface of tumor cells (*n* = 3). **(G, H)** CHF suppresses the IFNγ-triggered upregulation of JAK2, STAT1, and PD-L1, as well as phosphorylated STAT1 (*n* = 3). **(I)** Exogenous reconstitution of STAT1 in CHF-exposed cells partially restored the expression of JAK2 and PD-L1. Data are presented as means ± SD. *p < 0.05, **p < 0.01, ***p < 0.001, based on independent-samples *t*-test or one-way ANOVA.

T cell-mediated specific immune responses are important mechanisms for eliminating abnormal cells. To investigate the role of CHF in tumor immune responses, we first performed CD3^+^ and CD8^+^ IHC staining to evaluate the T cell profile of tumor-infiltrating immune cells in B16 xenograft tumors. We observed that the number of infiltrating CD3^+^ and CD8^+^ T cells in tumor tissues of CHF-treated mice was significantly increased compared with the control group (Figure 5A), indicating that CHF enhances the infiltration of CD3^+^ and CD8^+^ T cells in tumor tissues of B16 tumor-bearing mice. We also quantified the expression profiles of multiple key inflammatory cytokines, including TNFα, IFNγ, IL-2, and IL-1β, in the tumor tissues, as a critical approach to systematically evaluate the effector functions of intratumorally infiltrating immune cells. The results demonstrated that the expression levels of TNFα, IFNγ, IL-2, and IL-1β in tumor tissues from CHF-treated mice were significantly higher than those in the control group (Figure 5B). This observation strongly indicated that the tumor immune microenvironment of CHF-treated mice is most likely accompanied by more robust and activated effector functions of tumor-infiltrating immune cells.

PD-L1, an important immunosuppressive molecule expressed on the surface of tumor cells, inhibits the activation and effector functions of CD3^+^ and CD8^+^ T cells by binding to the PD-1 receptor. Tumor cells frequently evade T cell surveillance by overexpressing PD-L1. Therefore, we hypothesized that CHF might inhibit tumor immune escape by suppressing PD-L1. Moreover, previous studies have shown that the JAK2-STAT1 signaling axis is involved in regulating PD-L1 expression in human melanoma cell lines (27). Upon activation, JAK2 undergoes autophosphorylation, which in turn phosphorylates STAT1. Phosphorylated STAT1 forms dimers, translocates to the nucleus, binds to the gamma-activated sequence (GAS) element in the *CD274* gene promoter region, and ultimately drives high expression of PD-L1 protein. To investigate whether CHF inhibits melanoma immune escape through the JAK2-STAT1-PD-L1 pathway, we performed RT-qPCR and Western blot to detect changes in the mRNA and protein levels of the aforementioned pathway. Compared with the control group, the mRNA and protein levels of JAK2, STAT1, and PD-L1 were significantly decreased in the CHF-treated group (Figures 5C, D), suggesting that CHF may reduce PD-L1 transcription by inhibiting JAK2-STAT1 signaling, thereby affecting tumor immune responses. Given that PD-L1 is a membrane molecule localized on the surface of tumor cells, we also examined PD-L1 expression on the tumor cell surface by flow cytometry. The results showed that the level of PD-L1 on the membrane surface of melanoma cells was significantly reduced in the CHF-treated group (Figures 5E, F). These findings further confirm that CHF may relieve the immunosuppression of CD3^+^ and CD8^+^ T cells by abrogating PD-L1 expression on the surface of tumor cells. Considering that the JAK2-STAT1 signaling pathway is activated upon IFNγ stimulation, we performed RT-qPCR and Western blot to examine the expression of JAK2, STAT1, and PD-L1 under IFNγ stimulation. The results showed that the mRNA and protein levels of JAK2, STAT1, and PD-L1, as well as phosphorylated STAT1, were significantly upregulated under IFNγ-stimulated conditions in A375 cells (Figures 5G, H), indicating that IFNγ effectively activates the JAK-STAT signaling pathway and induces PD-L1 expression. In contrast, the levels of these molecules were markedly decreased in CHF combined with IFNγ treatment group (Figures 5G, H), supporting that CHF acts as a potential inhibitor targeting the JAK2-STAT1 signaling cascade. To define the specific role of STAT1 in CHF-mediated PD-L1 downregulation, we conducted a rescue experiment in CHF-treated cells. Notably, exogenous reconstitution of STAT1 in CHF-exposed cells partially restored the expression of JAK2 and PD-L1 (Figure 5I), confirming that CHF attenuates PD-L1 expression predominantly through specific suppression of STAT1. Collectively, these results demonstrated that CHF effectively inhibits IFNγ-induced activation of the JAK2/STAT1 signaling pathway and subsequently downregulates PD-L1 expression, which may represent an important molecular mechanism by which it relieves tumor immunosuppression and enhances anti-tumor immune responses.

### Metabolomic profiling of CHF in inhibiting melanoma

**Figure 6:**
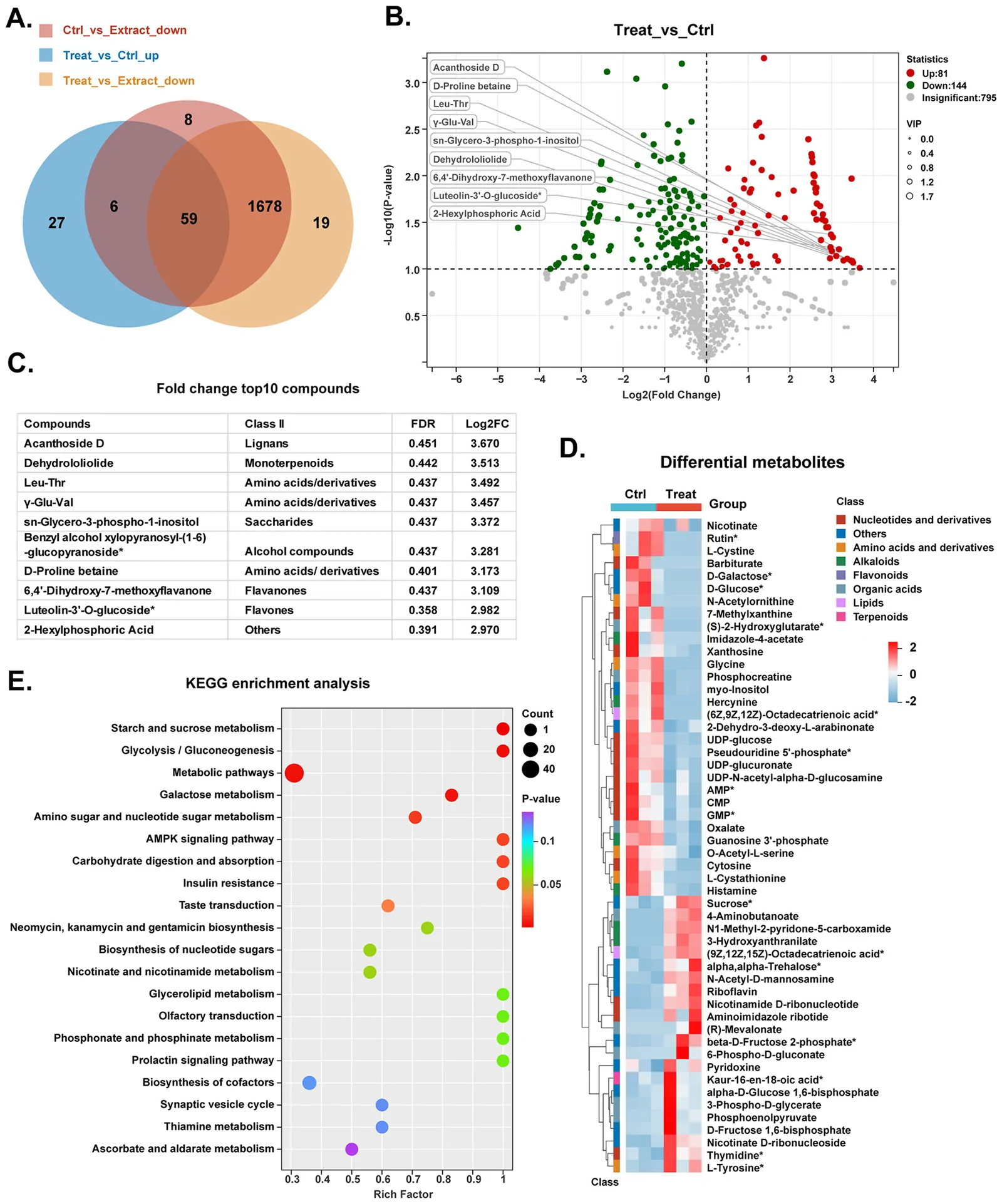
Metabolomic profiling of CHF in inhibiting melanoma. **(A)** Venn analysis of metabolites in each group. A total of 59 common intersecting metabolites were identified across the three groups. **(B, C)** Volcanic analysis of differential metabolites. The top 10 potential active components among the 59 common metabolites in CHF included Acanthoside D, Dehydrodoliolide, Leu-Thr, γ-Glu-Val, sn-Glycero-3-phospho-1-inositol, Benzyl alcohol xylopyranosyl-(1-6)-glucopyranoside, D-Proline betaine, 6,4'-Dihydroxy-7-methoxyflavanone, Luteolin-3'-O-glucoside, and 2-Hexylphosphoric Acid. **(D)** Functional clustering analysis of differential metabolites. **(E)** KEGG enrichment analysis of differential metabolites.

To further reveal the potential functional characteristics of CHF in inhibiting melanoma from a metabolic perspective, this study employed metabolomics technology to analyze the metabolic profile differences among the control group, the CHF treatment group, and the CHF extract group. Venn analysis showed that a total of 92 metabolites were significantly upregulated in the treatment group compared with the control group, 1,751 metabolites were significantly downregulated in the control group compared with the extract group, and 1,756 metabolites were significantly downregulated in the treatment group compared with the extract group. Among these, 59 metabolites represented the common intersection of the three comparisons (Figure 6A, Supplementary Table 1), suggesting that this set of differential metabolites may represent the potential core components responsible for the anti-cancer function of CHF. To further clarify the overall impact of treatment intervention on the metabolic profile, we performed differential analysis of metabolites between the treatment group and the control group. In the comparison between the CHF-treatment group and the control group, 81 upregulated and 144 downregulated metabolites were identified. Among the 59 potential active components of CHF, the top 10 included Acanthoside D, Dehydrodoliolide, Leu-Thr, γ-Glu-Val, sn-Glycero-3-phospho-1-inositol, Benzyl alcohol xylopyranosyl-(1–6)-glucopyranoside, D-Proline betaine, 6,4′-Dihydroxy-7-methoxyflavanone, Luteolin-3′-O-glucoside, and 2-Hexylphosphoric Acid (Figures 6B, C; Supplementary Figures S3, S4). These metabolites may play important roles in the anti-melanoma activity of CHF. Furthermore, we performed cluster analysis on the differential metabolites screened in Figure 6B. The results showed that these differential metabolites involved various categories of active components, mainly including nucleotides and their derivatives, amino acids and their derivatives, alkaloids, flavonoids, organic acids, lipids, terpenoids, etc. (Figure 6D). Among them, the changes in certain flavonoids and amino acid metabolites were consistent with the known antioxidant and anti-inflammatory activities of CHF. To further explore the biological functions and potential mechanisms of action of the differential metabolites, we performed KEGG pathway enrichment analysis. The results showed that several carbohydrate metabolism-related pathways were significantly enriched, including starch and sucrose metabolism, glycolysis/gluconeogenesis, galactose metabolism, and amino sugar and nucleotide sugar metabolism (Figure 6E). Other significantly enriched pathways included the AMPK signaling pathway and insulin resistance. These results indicate that CHF extract significantly regulates melanoma metabolism, primarily exerting its biological effects by modulating carbohydrate metabolism, energy homeostasis, and related signal transduction pathways.

### CHF enhances the therapeutic efficacy of PD-1/PD-L1 antibodies

**Figure 7:**
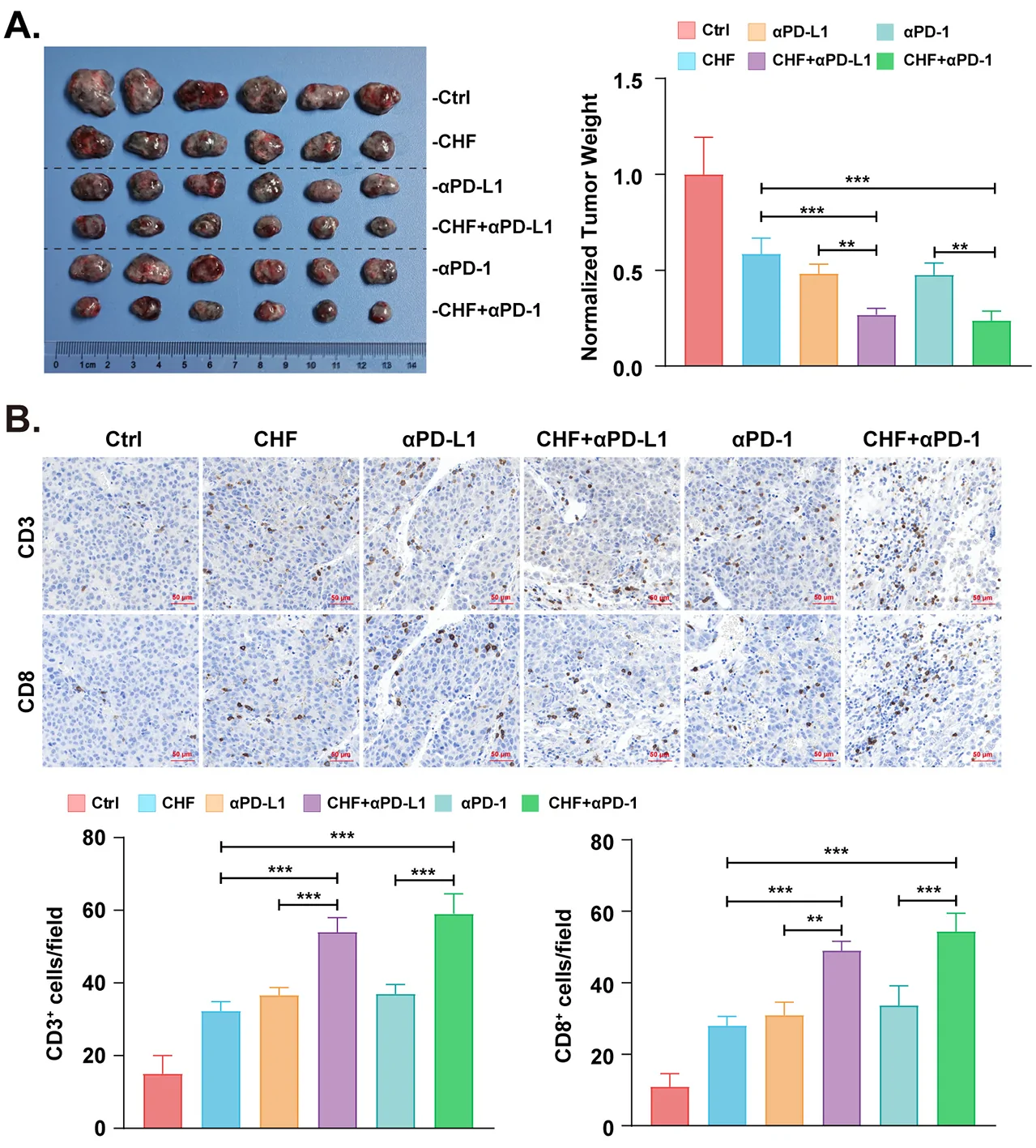
CHF enhances the therapeutic efficacy of PD-1/PD-L1 antibodies. **(A)** The combination therapy of PD-L1/PD-1 inhibitors with CHF more significantly inhibits the growth of mice xenograft tumors. **(B)** The number of infiltrating CD3^+^ and CD8^+^ T cells in the CHF combined with αPD-L1 (CHF+αPD-L1) and CHF combined with αPD-1 (CHF+αPD-1) groups is significantly higher than that in the respective monotherapy groups (αPD-L1 or αPD-1 alone) and the CHF-treated alone group. Data are presented as means ± SD. **p < 0.01, ***p < 0.001, based on independent-samples *t*-test or one-way ANOVA.

The above studies have demonstrated that CHF affects melanoma immune progression by suppressing PD-L1 expression. To further investigate whether it enhances the efficacy of immune checkpoint inhibitors, we performed combination therapy experiments of CHF with PD-L1/PD-1 inhibitors in mice. The results showed that compared with PD-L1/PD-1 inhibitor monotherapy, the combination with CHF significantly suppressed the growth of mouse xenograft tumors (Figure 7A), indicating that CHF further improves the anti-tumor efficacy of immune checkpoint inhibitors. To further explore the infiltration of CD3^+^ and CD8^+^ T cells in tumor tissues of each group, the CD3^+^ or CD8^+^ immunohistochemical staining was performed on the aforementioned specimens. The results showed that the number of infiltrating CD3^+^ and CD8^+^ T cells was increased in the CHF monotherapy group compared with the control group. Moreover, the infiltration numbers of CD3^+^ and CD8^+^ T cells in the CHF combined with αPD-L1 (CHF+αPD-L1) or αPD-1 (CHF+αPD-1) groups were significantly higher than those in the antibody monotherapy groups (αPD-L1 or αPD-1 alone) or the CHF monotherapy group (Figure 7B). These results indicate that CHF combined with immune checkpoint inhibitors enhances anti-tumor effects by synergistically increasing T cell infiltration into tumor tissues, further supporting the mechanism by which CHF enhances anti-tumor immune responses through modulating the tumor immune microenvironment.

## Discussion

**Figure 8:**
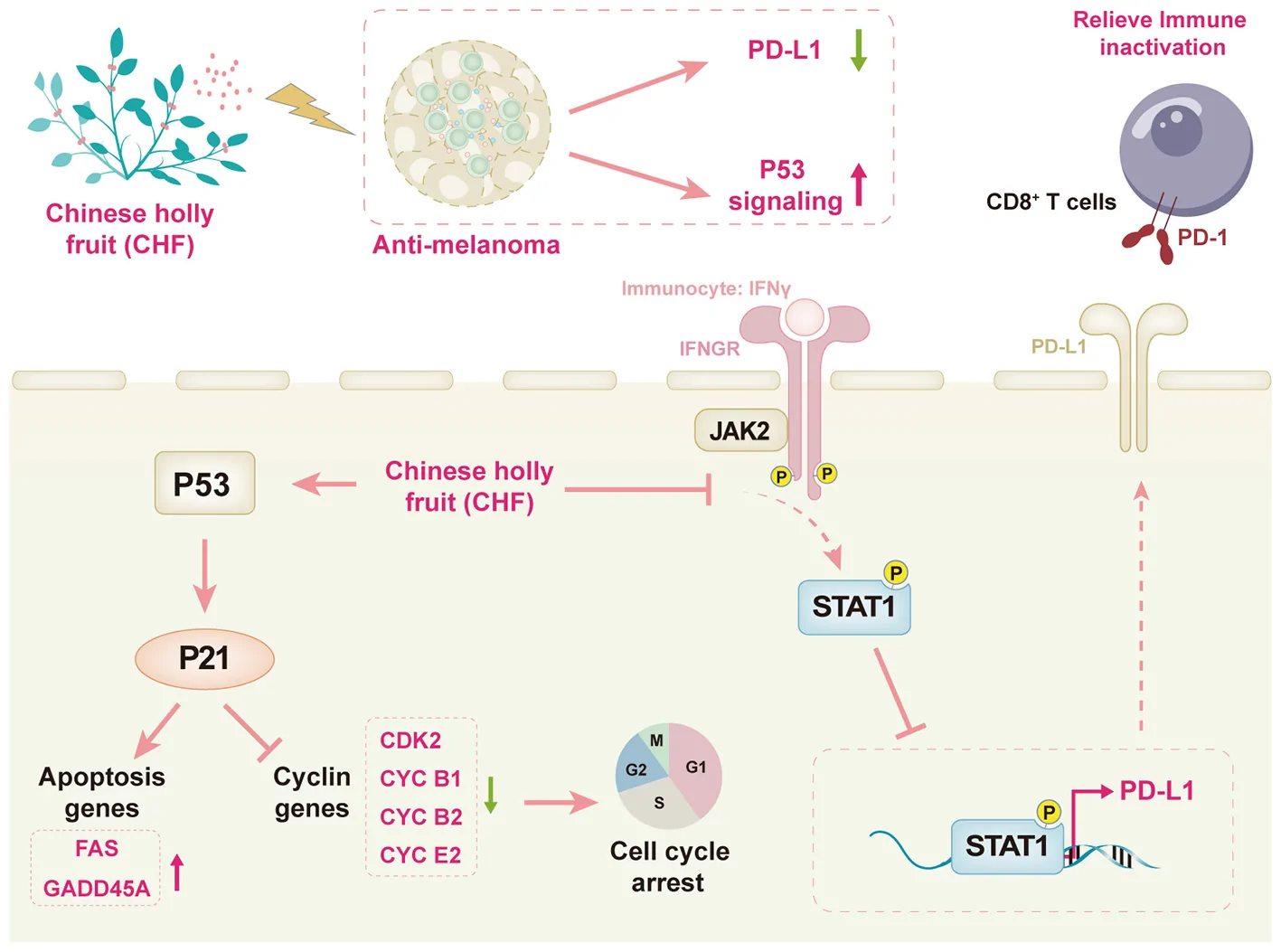
The anti-melanoma action of CHF. CHF promotes tumor cell apoptosis by upregulating apoptosis-related genes such as *FAS* and *GADD45A*. CHF arrests the cell cycle by activating the P53-P21 signaling pathway, and downregulates CDK2 as well as cyclins including Cyclin B1, Cyclin B2 and Cyclin E2. CHF downregulates PD-L1 expression by inhibiting the JAK2-STAT1 signaling pathway, thereby alleviating the immunosuppression of CD8^+^ T cells.

In this study, we reveal for the first time that the traditional Chinese herbal medicine holly (*Ilex cornuta Lindl. ex Paxt*) fruit (Chinese holly fruit, abbreviated as CHF) inhibits melanoma progression through multiple molecular mechanisms and characterize its immunomodulatory role within the tumor microenvironment. The proposed mechanistic model is as follows (Figure 8): CHF activates the P53-P21 signaling pathway, downregulates CDK2 and cyclins including Cyclin B1, Cyclin B2, and Cyclin E2, and induces cell cycle arrest. Meanwhile, CHF promotes tumor cell apoptosis by upregulating apoptosis-related genes such as *FAS* and *GADD45A*. Importantly, CHF also downregulates the expression of the immune checkpoint molecule PD-L1 by inhibiting the JAK2-STAT1 signaling pathway, thereby relieving the immunosuppression of CD8^+^ T cells and promoting their infiltration, activation, and recovery of cytotoxic function within the tumor site. Furthermore, Metabolomic profiling reveals that differential metabolites following CHF intervention are significantly enriched in glucose metabolism, energy metabolism, and the AMPK signaling pathway. In conclusion, CHF exerts its multifaceted antitumor effects against melanoma through dual mechanisms—molecular regulation and metabolic remodeling—as well as synergistic interactions between cell cycle control and the immune microenvironment. This pleiotropic mode of action aligns with the perspective emphasized in recent research on natural products: plant-derived compounds often function as multi-target modulators within disease-related signaling networks, rather than as conventional single-target inhibitors (28).

The inhibitory effect of CHF on the JAK-STAT signaling pathway and its regulation of PD-L1 expression are closely associated with the mechanisms of tumor immune escape. In the tumor microenvironment, activated CD8^+^ T cells release cytokines such as IFNγ, which induce PD-L1 expression on tumor cells via the JAK-STAT pathway (29). PD-L1 then binds to PD-1 on the surface of T cells, suppressing T cell activation and function, thereby facilitating immune escape. In this study, we observed that treatment with CHF significantly reduced the levels of JAK2 and STAT1, inhibited PD-L1 expression at both the transcriptional and protein levels, and markedly increased the infiltration of CD3^+^ and CD8^+^ T cells in tumor tissues. These results demonstrated that CHF attenuates the immunosuppressive capacity of tumor cells against CD8^+^ T cells by inhibiting the JAK2-STAT1 pathway, supporting our proposition that CHF acts as an immune escape intervention agent. In addition, knockdown P53 significantly attenuated the upregulation of P21 induced by CHF and partially rescued the expression of CDK2, confirming the central role of the P53-P21 pathway in the antitumor activity of CHF. Concurrently, exogenous reconstitution of STAT1 in CHF-exposed cells partially restored the expression of JAK2 and PD-L1, confirming that CHF attenuates PD-L1 expression predominantly through specific suppression of STAT1. Accumulating evidence indicates that JAK2 inhibitors can indirectly activate P53 via multiple mechanisms, including downregulation of MDM2 and induction of DNA damage (30). This suggests that CHF-activated P53 may synergize with its inhibition of JAK2-STAT1 signaling, thereby enhancing and prolonging its antitumor effects. In addition to the two major pathways mentioned above, CHF also plays a role in metabolic regulation. In this study, metabolomic profiling revealed that differential metabolites following CHF intervention were enriched in the AMPK pathway and associated glucose and energy metabolism pathways, suggesting that CHF may further suppress melanoma progression through metabolic remodeling. AMPK is not only a cellular energy sensor but has also been reported to form a regulatory loop with P53. AMPK can enhance the transcriptional activity of P53, while P53 can in turn upregulate the expression of AMPK-related metabolic genes (31). This bidirectional crosstalk effectively couples metabolic reprogramming with cell cycle arrest, thereby synergistically potentiating the antiproliferative effects of CHF on tumor cells.

Although current JAK inhibitors (e.g., tofacitinib, ruxolitinib) for melanoma have shown certain therapeutic efficacy, they are associated with side effects such as infection risk (32, 33), dyslipidemia (34), and anemia (35), and drug resistance is increasingly prominent. And resistance to JAK inhibitors has been confirmed to be associated with loss of P53 function (36). Combination therapy has become an important strategy to enhance efficacy and delay drug resistance; however, safe and effective combination regimens still need to be explored. Our study found that the combination treatment exhibits superior inhibition of xenograft tumor growth, and the number of infiltrating T cells was significantly higher in combination groups than that in monotherapy groups. This finding suggests that CHF may serve as a potential combination therapeutic agent that enhances the anti-tumor efficacy of immune checkpoint inhibitors. Although clinical experience with CHF is currently lacking, our findings provide theoretical support for its combined application in melanoma immunotherapy.

Despite these promising findings, several limitations of the present study should be acknowledged. CHF has a complex chemical composition, and the specific monomeric components responsible for its antitumor and immunomodulatory activities have not yet been identified. In the current study, we have successfully identified a panel of candidate bioactive monomers present in CHF via untargeted metabolomic profiling, and we have specifically listed the top 10 representative active components with the highest abundance and research potential. At this stage, we are systematically conducting further investigations to fully characterize the underlying functional active substances in CHF, by integrating high-precision mass spectrometry, multi-dimensional chromatographic separation and targeted metabolomic validation strategies. We sincerely anticipate that we can thoroughly elucidate these critical scientific questions and present the complete mechanistic landscape in our follow-up dedicated studies. The current study is still limited to cellular experiments and animal models, and only preliminarily confirms that CHF can inhibit melanoma progression and interfere with its immune escape process. Its specific efficacy, pharmacokinetic characteristics *in vivo*, and long-term safety remain to be further elucidated. Moreover, whether CHF is equally effective against melanomas with low or negative PD-L1 expression, as well as its differential sensitivity across distinct melanoma subtypes, also requires further clarification. We believe that with in-depth research on molecular mechanisms, signaling networks and multi-omics analysis, the exact mechanism underlying the anti-tumor effect of CHF will be revealed.

## Data Availability

The raw sequence data reported in this paper have been deposited in the Genome Sequence Archive in National Genomics Data Center, China National Center for Bioinformation (GSA-Human: HRA019502).
